# A Scoping Review of Eating Disorder Prevention and Body Image Programs Delivered in Australian Schools

**DOI:** 10.3390/nu17132118

**Published:** 2025-06-26

**Authors:** Sharri Sarraj, Sophie L. Berry, Amy L. Burton

**Affiliations:** 1 School of Psychology, The University of Sydney, Sydney, NSW 2000, Australia; 2 Graduate School of Health, University of Technology Sydney, Sydney, NSW 2000, Australia

**Keywords:** eating disorders, body image, prevention, schools, children, adolescents

## Abstract

Background: Eating disorders (EDs) are complex conditions with significant psychological, physical, and economic impacts, prompting national calls to prioritize ED prevention. Despite numerous prevention programs being implemented in Australian schools, no review to date has systematically mapped their scope, design, and outcomes. Aims: This scoping review aimed to map the current landscape of school-based ED prevention programs conducted in Australia. The review focused on their methodological features, participant and school characteristics, data collected, and key findings. Method: Four electronic databases (MEDLINE, PsycINFO, EMBASE, and Scopus) were searched for relevant papers published from 2010 to February 2025. Studies were included if they reported on a school-based ED prevention program targeting Australian students. Data were extracted and narratively synthesized. Results: A total of 23 studies were identified, representing a range of universal and selective prevention programs. Programs varied in design, delivery, and target populations, with most focusing on students in Grades 7–8. Universal media literacy programs like Media Smart showed good outcomes for boys and girls, while several selective programs demonstrated improvements in body image for girls. Interventions targeting boys or using mindfulness approaches often lacked effectiveness or caused unintended harm. Major gaps in the literature include a lack of qualitative research, limited long-term follow-up, and minimal focus on protective factors. Conclusion: While a range of ED prevention programs have been trialed in Australian schools, few have been rigorously evaluated or demonstrated sustained effectiveness. There is a need for developmentally appropriate, gender-sensitive, and culturally inclusive prevention efforts in schools. Future research should use diverse methods, include underrepresented groups, assess long-term outcomes, integrate broader sociocultural factors shaping students’ environment, and consider enhancing protective factors.

## 1. Introduction

Eating disorders (EDs) are complex and often chronic conditions associated with significant psychological (e.g., anxiety, mood, and substance use) and physical comorbidities [1], reduced health-related quality of life [2], and elevated mortality rates [3]. In Australia, the economic burden of EDs was estimated at AUD20.8 billion in 2023 [4], underscoring the urgent need for systemic reform. In response, the Australian National Eating Disorders Collaboration (NEDC) released a ten-year national strategy advocating for a stepped care model that situates the prevention of EDs as the first component [5].

The NEDC defines prevention as “actions, programs, or policies that aim to reduce modifiable risk factors for eating disorders, and/or bolster protective factors, to reduce the likelihood that a person will experience an eating disorder” [5]. The NEDC further recognizes schools as the “ideal position” to deliver such strategies, particularly those targeting well-established risk factors, which often emerge in adolescence [6]. Alarmingly, ED prevalence rates and body image concerns are highest among school-aged children and youth [4,7,8]. In a national surveillance study of early-onset EDs in Australia, children aged 5–13 years presented with prolonged symptom duration, significant weight loss, and high rates of medical compromise requiring refeeding [9]. Given that adolescence is the peak period of ED onset—and the age of onset is becoming increasingly younger—combined with the severity and burden of ED pathology, we echo the call to action raised by Yager [10] “we need prevention now, more than ever” (p. 729).

### 1.1. School-Based Prevention Programs

Indeed, three decades of international research efforts have focused on the development and implementation of school-based ED prevention programs [11]. School-based programs tend to focus on reducing individual and socio-environmental risk factors for ED, such as body dissatisfaction, appearance-ideal internalization, and the negative impacts of peers and the media on these factors, and enhancing protective factors such as self-esteem [11]. Programs may be universal, targeting all students, or selective, focusing on at-risk groups such as adolescent girls with heightened body image concerns [12].

Several recently published systematic reviews have examined the effectiveness of school-based ED prevention programs on outcomes such as core ED psychopathology [13,14], body image [15], and mental health indicators [16]. Berry et al. [13] found that media literacy and dissonance-based interventions produced small to medium effects on ED symptomatology immediately after the intervention, with follow-up results ranging from small negative to moderate positive effects. However, the long-term effectiveness of these programs remains inconsistent, especially among school-aged children, where sustained reductions in ED symptomatology have not been reliably observed [13]. Another systematic review found that universal prevention programs led to improvements in body esteem, self-esteem, and appearance-ideal internalization, but not body dissatisfaction, among children [15]. When also considering selective prevention programs, Pursey et al. [14] reported a general trend toward reduced risk factors for EDs, particularly improvements in body image; however, the effects varied significantly across studies. While these reviews provide valuable insights, none have focused exclusively on school-based ED prevention programs implemented in Australia, where cultural and educational contexts may influence program design, delivery, and outcomes.

### 1.2. Rationale for the Current Review

Taken together, these findings suggest that schools hold considerable promise as a setting for ED prevention, offering broad access to young people during critical developmental windows [17]. While current programs show encouraging short-term outcomes, such as improvements in body image and self-esteem, their long-term effectiveness remains inconsistent. Nonetheless, with further refinement and investment in developmentally appropriate, evidence-based approaches, school-based programs can play a meaningful role in ED prevention efforts.

Despite the growing number of prevention programs implemented in Australia, no review to date has comprehensively mapped the scope, design, and outcomes of school-based ED prevention programs in Australia. Addressing this gap is essential to inform future research, policy, and practice. A scoping review methodology is suitable for this purpose as we aim to map the existing research base and identify gaps in knowledge to inform future Australian research priorities [18].

The research questions guiding our scoping review were as follows:

1. What are the key methodological features of the school-based prevention programs (e.g., universal or selective, content, format, mode of delivery, and follow-up)?;
2. What are the demographic and general characteristics of the schools and participants involved in these studies (e.g., age, grade, gender, ethnicity, and location)?;
3. What outcome measures or data have been collected in these studies?;
4. What are the key findings of available prevention programs based on reported outcome measures?

## 2. Methods

This scoping review was guided by the JBI methodology for scoping reviews [19], and the Preferred Reporting Items for Systematic Reviews and Meta-Analyses extension for scoping reviews (PRISMA-ScR) [20]. A copy of the PRISMA checklist is included as a Supplementary File. This review was supported by funding from the Graduate School of Health, Faculty of Health, University of Technology Sydney.

### 2.1. Protocol and Registration

An a priori protocol was developed and prospectively registered with the Open Science Framework (OSF) available at https://osf.io/kvade (accessed on 28 October 2024). No major deviations from the protocol occurred, aside from a post hoc decision to limit the search to studies published from 2010 onwards. This decision was based on team consensus that earlier studies were often outdated in terms of theoretical frameworks and methodologies, or had already been synthesized in previous reviews.

### 2.2. Eligibility Criteria

To meet the inclusion criteria, studies must target Australian school-aged children or adolescents aged 4–18 years. All types of schooling were considered (e.g., public, private, mainstream, single-sex, and specialized schools). Prevention programs must aim to target ED symptomology and/or modifiable risk factors such as disordered eating, body image, body dissatisfaction, and shape and weight concerns. The intervention may be delivered in any format if it is conducted within a school setting (e.g., computer program, clinician-led, peer-led, or teacher-delivered). Included articles must present original research, whilst reviews, meta-analyses, opinion papers, and conference abstracts were excluded. Finally, articles had to be published from 2010, and accessible in full-text and available in English due to resource limitations.

### 2.3. Search Strategy

An initial exploratory search of PsycINFO was conducted to identify relevant review articles and inform the development of a comprehensive search strategy. Keywords and index terms from these articles were refined in consultation with an experienced university librarian. The final search strategy was developed for PsycINFO (OVID; see Appendix A) and adapted for three additional databases, MEDLINE, EMBASE, and Scopus. The initial search was conducted in October 2024 and re-run in February 2025. Reference lists of included studies and relevant reviews were also screened using citation chaining to identify additional eligible studies.

### 2.4. Study Selection Process

The identified papers were imported into Covidence software, https://app.covidence.org (accessed on 30 October 2024), where they were de-duplicated and screened against the inclusion criteria. At the title and abstract level, one reviewer (SS) screened all articles, whilst a second reviewer (SLB) independently screened 20% of the papers to verify accuracy. The reviewers achieved 98% agreement at this stage, with any disagreements resolved through team discussion. At the full-text screening stage, both reviewers (SS and SLB) independently assessed all articles against the inclusion criteria and reached 95% inter-rater agreement.

### 2.5. Data Charting and Presentation

A data-charting table was created in Covidence and used to extract data items from included papers. This included bibliographical information, study aims and design, population characteristics, school characteristics, intervention characteristics, outcome measures collected, and key findings. For this review, co-educational and boys-only prevention programs were classified as universal, whilst girls-only programs were classified as selective prevention. This classification was guided by the Institute of Medicine (IOM) framework, which defines universal prevention as targeting the general population regardless of risk, and selective prevention as targeting subgroups at elevated risk [21]. Given that boys are not considered a high-risk group in the eating disorder literature, and boys-only programs were delivered in general school settings without screening for risk, which aligns with the IOM definition. Extracted data are presented in tabular format below and accompanied by a descriptive synthesis of results.

## 3. Results

### 3.1. Search Results

**Figure 1:**
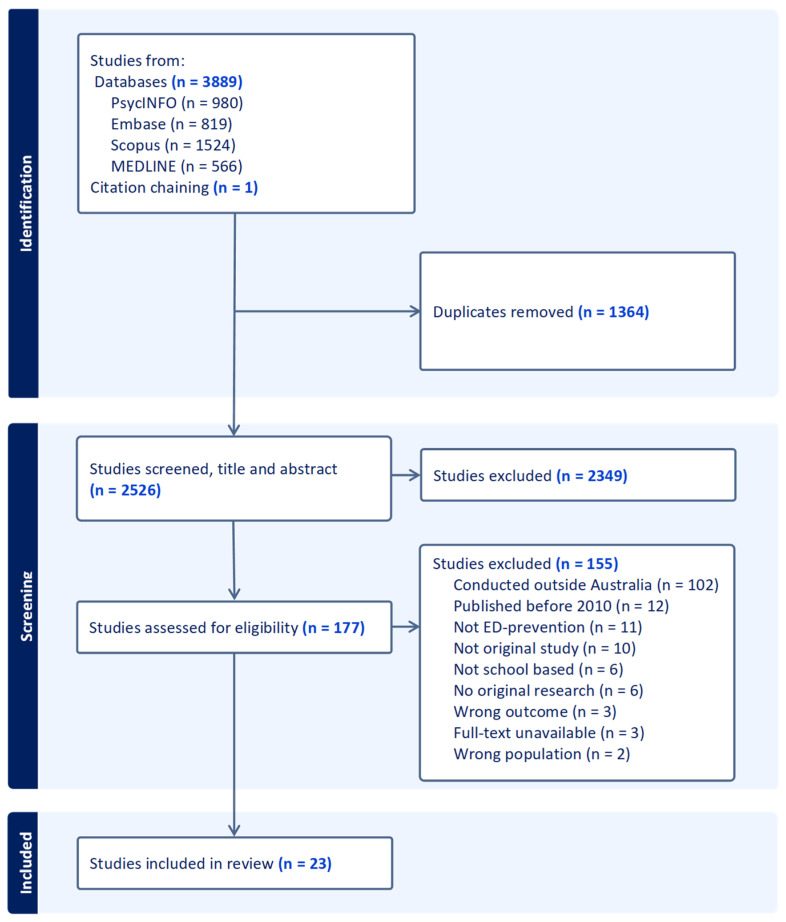
PRISMA flowchart of the study selection process.

The screening and study selection process is illustrated in the PRISMA flowchart in Figure 1. A total of 3889 records were identified through database searches, and one record through citation chaining. Of these articles, 1364 were removed as duplicates. After initial screening of 2526 articles, 177 papers were examined at the full-text level, and 155 of these were excluded. A total of 23 studies from 22 articles were included in the final review, as one article is a multi-study paper. Additionally, one RCT was accompanied by a secondary mediator analysis and moderator analysis, which were included due to their relevance to the original RCT intervention.

### 3.2. Characteristics of Included Studies

Table 1 summarizes key characteristics of the included studies, including each intervention’s aim and design, sample size, school setting and location, participant demographics, and method of delivery.

### 3.3. Synthesis of Results

Table 2 provides a summary of the intervention characteristics, duration, and follow-up, data collected, and key findings from each source of evidence. The narrative synthesis below is structured around the four guiding research questions, with findings grouped by the program approach.

#### 3.3.1. What Are the Key Methodological Features of the School-Based Prevention Programs?

A total of 23 studies were included in this review. The most common study design was randomized controlled trials (RCTs; *n* = 11), followed by quasi-experimental studies (*n* = 10) and secondary analyses of RCTs (*n* = 2). These studies evaluated a range of prevention programs targeting ED risk factors, including cognitive dissonance-based interventions (*n* = 3), media literacy programs (*n* = 6), multi-component interventions addressing multiple ED risk factors (*n* = 8), mindfulness-based approaches (*n* = 3), self-esteem enhancement programs (*n* = 2), and healthy lifestyle promotion (*n* = 1).

In terms of prevention level, twelve studies implemented universal prevention programs in co-educational settings. Three studies delivered body image programs exclusively targeting boys, while seven studies focused on selective prevention for girls. Two studies included both a selective (girls-only) arm and a universal (co-educational or boys-only) comparison group. Delivery was primarily facilitated by external professionals (*n* = 16), followed by teachers (*n* = 6), peers (*n* = 1), and video presentations (*n* = 1). Session lengths ranged from brief 15 min modules to 90 min weekly sessions, with most programs delivered over 3 to 10 sessions. Follow-up periods were similarly variable: seven studies included a 3-month follow-up, while others extended to 6, 9, or 12 months. Four studies did not include any follow-up assessment.

#### 3.3.2. What Are the Demographic and General Characteristics of the Schools and Participants Involved in These Studies?

The 23 studies were conducted across more than 74 schools in five Australian states, with the majority based in Victoria and South Australia, and additional representation from.

Queensland, New South Wales, and Western Australia. Most schools were situated in urban or suburban areas, although specific classifications (e.g., rural or regional) were not consistently reported.

Participants were primarily students in Grades 7–8 (*n* = 14), followed by those in Grades 9–12 (*n* = 6), and Grades K–6 (*n* = 3). Grade level was not reported in two studies. Ethnicity and cultural background were also inconsistently reported across studies. Where data were available, most samples were predominantly Caucasian, with proportions ranging from 62% to 84%. Several studies reported participants’ country of birth as a proxy for cultural background, with between 64% and 90% of participants born in Australia.

#### 3.3.3. What Outcome Measures or Data Have Been Collected in These Studies?

A wide range of outcome measures was used to assess program effectiveness and feasibility. Data collection instruments that were commonly used to assess ED risk and related constructs included the Eating Disorder Examination Questionnaire (EDE-Q; 10 studies; [44]), the Dutch Eating Behavior Questionnaire (DEBQ; 9 studies; [45]), and the Sociocultural Attitudes Towards Appearance Questionnaire (SATAQ; 13 studies; [46]), particularly the thin-ideal internalization subscale. Thirteen studies also evaluated program implementation and feasibility via teacher or student feedback. These included measures of student and teacher acceptability, fidelity to program content, and perceived value. A subset of studies explored additional behavioral and contextual factors, such as screen time, physical activity, fruit and vegetable intake, and attitudes toward supplement and steroid use. These exploratory measures were more common in programs with a broader health promotion or media literacy focus. Notably, no studies employed structured clinical interviews or diagnostic tools to assess ED onset as an outcome variable.

#### 3.3.4. What Are the Key Findings of Available Prevention Programs?

##### Dissonance-Based Interventions

Of the 23 prevention studies included in this review, three studies utilized a cognitive-dissonance approach to target appearance-ideal internalization [22,30,43]. Whilst all three interventions drew from *The Body Project* [47], there was variability in the population focus, session structure, and delivery method among studies.

One study by Atkinson and Wade [22] employed an RCT design to compare outcomes of a three-session dissonance-based program, a mindfulness program, and a control group for adolescent girls in grades 10–12. When delivered by an expert facilitator, the dissonance-based program led to a reduction in sociocultural pressures at the 6-month follow-up [22]. Whilst dissonance programs often target thin-ideal internalization in at-risk girls, Yager et al. [43] evaluated a program (*Goodform*) specifically designed to address the muscular ideal and discourage anabolic steroid and supplement use in adolescent boys. Regardless of study conditions, boys’ muscularity dissatisfaction, appearance pressures, and positive attitudes towards steroid use all increased over the approximately eight-week study period.

A third study by Kristoffersen et al. [30] explored the acceptability and feasibility of a video-based dissonance intervention compared to a self-compassion video among adolescents aged 15–17. While more boys than girls reported applying dissonance-based techniques at one-week follow-up, boys in the dissonance group also experienced a significant reduction in positive mood.

##### Media Literacy Interventions

Six studies employed a media literacy approach, which aims to enhance students’ critical thinking about appearance-focused content and media-driven appearance ideals. Among these, four studies evaluated the *Media Smart* program, a teacher-delivered intervention designed to target media internalization (investment in appearance-ideals promoted in the media) [48]. In an initial pilot study, *Media Smart* was found to reduce feelings of ineffectiveness and weight-related peer teasing among both boys and girls post-intervention, with sustained improvements in peer teasing reported at 6-month follow-up [38]. In a subsequent, RCT, *Media Smart* was compared to two alternative school-based programs—*Life Smart*, a healthy lifestyle and obesity prevention program, and *HELPP*, a multi-risk factor program adapted from *Happy Being Me* (HBM)—as well as a no-intervention control group [39]. Results showed that girls who completed *Media Smart* reported fewer eating concerns than those in the *HELPP* group at 6 months follow-up, and fewer weight and shape concerns than those in the *Life Smart* group at 12-month follow-up [39]. Boys who participated in *Media Smart* demonstrated improvements in body dissatisfaction, media internalization, weight-related peer teasing, and perfectionism, with sustained benefits in media internalization and depression at both 6- and 12-month follow-ups [39]. In contrast, boys in the *Life Smart* program experienced increased media internalization and higher levels of depression at follow-up, despite some initial improvements in body dissatisfaction [39]. To better understand for whom the programs were most effective, Wilksch et al. [40] used moderation analysis, and their results indicated that students with higher baseline shape and weight concerns who completed either the *Life Smart* or *HELPP* interventions reported increased eating concerns and meal skipping at follow-up compared to those who completed Media Smart [40]. Mediator analysis conducted by Wade et al. [41] further identified media internalization as a key mechanism underlying improvements in body image across both *Media Smart* and *Life Smart* programs.

Two additional studies extended the traditional media literacy approaches to the unique context of social media [26,33]. Girls who participated in the *Boost* social media literacy program exhibited improvements in body esteem, dietary restraint, and media literacy compared to control participants [33]. Similarly, Gordon et al. [26] assessed the universal *SoMe* program in a large multi-school RCT. Gender-specific effects were observed where girls in the *SoMe* group reported significantly lower levels of dietary restraint at 6-month follow-up, while boys showed increased self-esteem but also a greater drive for muscularity at 12-month follow-up [26].

##### Multicomponent Prevention Programs

Multicomponent prevention programs typically target one or more modifiable risk factors and may integrate different theoretical approaches to ED prevention. Three studies included in this study evaluated *HBM*, which addresses both individual ED risk factors via a media literacy approach as well as the influence of the peer environment on body image [24,34,35]. Improvements in thin-ideal internalization, body comparisons, appearance-related conversations with peers, body satisfaction, dietary restraint, and self-esteem were observed when *HBM* was delivered as a selective intervention with adolescent girls [35]. To better understand the active components of the program, McLean et al. [34] conducted a dismantling study that separated and compared the media literacy and appearance comparison content of *HBM*. Girls in the *HBM*-comparison group experienced improvements in bulimic symptoms and thin-ideal internalization, while the *HBM*-media group showed some improvements in appearance comparisons [34]. Importantly, Dunstan et al. [24] found that *HBM* was comparably effective for girls when delivered in a co-educational context.

Building on the *HBM* framework, *Dove Confident Me* (DCM) was developed through co-design with adolescents, educators, and experts to enhance relevance and engagement. In an evaluation, Forbes et al. [25] found that adolescent girls who participated in *DCM* reported higher sociocultural pressures compared to the control group immediately post-intervention, but also demonstrated greater reductions in social comparisons by three-month follow-up. However, qualitative feedback from teachers indicated low student engagement and limited perceived efficacy, prompting revisions to the program content [25]. Despite these modifications, girls continued to report elevated sociocultural pressure and thin-ideal internalization post-test, the latter also maintained at follow-up [25].

Another selective prevention program, *Y’s Girl*, addressed topics such as cultural beauty practices, body positivity, communication skills, positive self-talk, and media literacy [36]. Grade 6 girls in the *Y’s Girl* group experienced significant improvements in body satisfaction and self-esteem, and reductions in body size discrepancy, thin-ideal internalization, and body comparisons, particularly amongst girls with initially high levels of body comparison and low self-esteem [36].

The *Healthy Me* program adopted a gender-sensitive approach by delivering sessions separately to boys and girls, addressing the distinct sociocultural pressures each gender faces [32]. Both boys and girls reported increased body and muscle esteem at follow-up [32]. Additionally, boys demonstrated higher physical activity levels and reduced endorsement of masculine gender norms, while girls showed improvements in fruit and vegetable intake [32].

Lastly, the *Life Smart* program was a mixed-gender universal program targeting multiple obesity and ED risk factors by promoting a holistic approach to health [37]. Student feedback indicated that boys rated the healthy eating topic most favorably, while girls preferred the sleep and exercise lessons [37]. Further, girls in the *Life Smart* group experienced improved shape and weight concern at post-intervention, while boys showed a small to moderate increase in physical activity [37].

##### Mindfulness-Based Programs

Three studies evaluated mindfulness-based prevention programs delivered universally to high school students. Each program utilized mindfulness strategies and practices aimed to address transdiagnostic risk factors purported to underpin a range of psychopathologies, including EDs. Whilst there was high acceptability of the *.b Mindfulness in Schools* program amongst students and teachers, no improvements were found in psychological outcomes at post-intervention or follow-up [27]. Indeed, higher levels of anxiety were reported by boys, and those with low baseline shape and weight concern and low baseline depression in the intervention group compared to the control [27]. The authors hypothesized that the mindfulness concepts may be developmentally inappropriate for younger adolescents in the study and that longer sessions with longer mindfulness practice could improve outcomes. Subsequently, Johnson and Wade [28] piloted the *Mindfulness Training for Teens* program, which is closely modeled on adult mindfulness programs. There were moderate improvements in depression and anxiety for Year 10 students compared to the control at follow-up, but no changes in weight and shape concerns [28]. A subsequent RCT tested a revised version of the program with shorter sessions and the removal of a 10 min break [29]. Again, no significant improvements were found in psychological outcomes compared to the control group. Notably, an age-related effect emerged where Year 8 students in the intervention group reported declines in mindfulness and wellbeing at three-month follow-up, while Year 10 students showed no significant changes over time [29].

##### Self-Esteem Enhancement

This review identified two studies that focused on improving self [31] and body esteem [23]. Adolescent boys 11–15 years old did not experience any improvements in body image or body satisfaction after a three-lesson self-esteem program [31]. In a younger population of children aged 5–8, Damiano et al. [23] evaluated the teacher-led *Achieving Body Confidence for Young Children* (ABC-4-YC) intervention. The pilot study found significant improvements in children’s body esteem following the three-lesson program [23].

##### Healthy Lifestyle Promotion

One study in this review utilized a healthy lifestyle promotion approach to enhance body satisfaction in a cohort of adolescent boys [42]. The *ATLAS* program educates boys on drug and supplement use, the benefits of strength training, and sports nutrition using a peer-facilitated model. While the program led to small, non-significant improvements in both functional and esthetic body image satisfaction, qualitative feedback indicated that students found the content on avoiding anabolic steroids and drugs more engaging than appearance-focused material [42].

## 4. Discussion

This scoping review sought to identify and describe school-based ED prevention programs implemented in Australia, with a focus on their methodologies, participants, outcomes, and findings. Overall, the review revealed that while a range of distinct universal and selective programs have been trialed, only a small number of programs have been evaluated across studies, and few have demonstrated sustained effectiveness across genders and developmental stages. These results underscore the need to critically examine not only what programs are being implemented, but also how, for whom, and under what conditions they are most effective.

The review identified a predominance of quantitative methodologies, often examining program feasibility or efficacy. Demographic reporting across the studies was markedly limited, significantly constraining the ability to assess sample diversity and generalizability. Only three studies reported student ethnicity, while 61% omitted cultural or ethnic data entirely and 26% reported only place of birth, providing minimal insight into the cultural composition of the samples. The majority of participants across the reviewed studies were in Grades 7–8, aligning with evidence suggesting that early adolescence (age 12–13) is a critical window for effective ED prevention [49]. Programs targeting this age group may be more impactful due to the developmental timing of body image concerns and the onset of sociocultural pressures. This contrasts with earlier findings by Stice et al. [50] which suggested greater effectiveness among older adolescents. The current review supports the shift toward earlier intervention, particularly when programs incorporate media literacy, self-esteem building, and peer influence components. However, the limited number of studies involving younger children or older teens highlights a need for more age-diverse research to determine how prevention strategies can be tailored across developmental stages.

Among the programs reviewed, *Media Smart* emerged as an effective universal prevention program. The program was associated with improvements in body dissatisfaction, media internalization, and depressive symptoms among boys, and reductions in eating concerns and weight and shape dissatisfaction among girls [39]. These findings align with meta-analytic evidence from Le et al. [51], which identified media literacy as the only approach to consistently yield small to moderate effect sizes in co-educational settings. Importantly, *Media Smart* also supports the feasibility of teacher-led delivery, which may enhance program scalability. However, the broader literature presents mixed findings regarding the effectiveness of teacher-led versus researcher-led implementation [14,15,52], and many teachers report lacking the training and institutional support necessary for confident delivery [53]. As suggested by surveyed teachers, a multimodal delivery model combining teacher facilitation with external expertise may offer a more sustainable and scalable solution [53].

Gender-specific patterns in program outcomes were also evident. Universal programs such as *SoMe* [26] and *Healthy Me* [32] demonstrated differential effects, with girls benefiting from reduced dietary restraint and healthier eating behaviors, while boys showed increased self-esteem but also, in some cases, a heightened drive for muscularity. These findings underscore the importance of gender-sensitive content and delivery formats. Selective programs targeting adolescent girls, such as *Happy Being Me* [24,34,35] and *Y’s Girl* [36] consistently improved body image outcomes and self-esteem, particularly among those with higher baseline vulnerability. In contrast, interventions designed specifically for boys, such as *Goodform* [43] and *Self-esteem and Healthy Body Image Program* [31], failed to produce significant improvements in body image or related behaviors. In some cases, participation was linked to unintended negative effects, such as reduced positive mood or increased appearance pressures.

While universal programs are often critiqued for yielding non-significant statistical outcomes, Yager [10] argues that such programs should not be dismissed when they are theoretically grounded and show no evidence of harm. This perspective is particularly relevant in school settings, where educators may rely on unvetted or improvised resources to deliver well-intentioned body image lessons. However, the presence of iatrogenic effects in some programs challenges the assumption that all prevention is benign and highlights the need for careful program selection and monitoring.

### 4.1. Clinical and Theoretical Implications

This review highlights several clinical and theoretical implications in the current landscape of school-based ED prevention in Australia. First, many programs continue to adopt an individual-level focus, targeting proximal risk factors such as body dissatisfaction and thin-ideal internalization, while underemphasizing broader sociocultural determinants. As Piran [54,55] have argued, prevention efforts that neglect the influence of systemic factors—such as gender norms, socioeconomic status, and cultural marginalization, risk oversimplifying the etiology of body image concerns and disordered eating. This critique has gained increasing interest due to the divergent etiological pathways between genders and the gendered outcomes evident in this study [56]. In a qualitative paper, Atkinson et al. [57] identified a pressing need to shift the field’s focus from individual and lower-level sociocultural factors to higher-level systems that shape appearance ideals and body image concerns for young people. For example, broader cultural shifts in masculine gender roles add further complexity to our understanding of how boys experience and respond to appearance pressures. Programs that fail to account for these evolving sociocultural dynamics may struggle to engage male participants meaningfully or produce lasting change.

Second, a critical theoretical and methodological issue in ED prevention research is the distinction between risk reduction and true prevention. Most of the studies included in this review were appropriately framed as risk reduction trials, focusing on proximal outcomes such as body dissatisfaction, dietary restraint, and thin-ideal internalization. While these are important targets, they do not equate to the prevention of clinically diagnosable EDs. As Becker [58] argues, the medical standard for prevention requires evidence of reduced onset of EDs, which demands large samples, long-term follow-up, and diagnostic interviews—conditions rarely met in school-based research. Stice et al. [59] further highlight the statistical challenges of detecting differences in ED onset due to the low base rate of these disorders and the reduced power of dichotomous outcome analyses. Notably, no studies employed diagnostic interviews or assessed ED onset as an outcome, nor included follow-up periods beyond 12 months. This limits the ability to draw conclusions about the long-term or clinical impact of these programs.

Third, the field remains heavily focused on risk reduction, with minimal attention to protective factors, as only one study [23] explicitly targeted self-esteem enhancement. As Levine and Smolak [60] argue, protective factors can buffer against a range of risks. The near-total absence of such approaches represents a missed opportunity to shift from a deficit-based model to one that fosters resilience and wellbeing. A recent exception is the *Embrace Kids Classroom Program* (EKCP), a school-based, positive psychology intervention targeting self-esteem, mindfulness, and body appreciation [61]. While showing positive impacts on self-compassion, the program faced some resistance from school communities due to its inclusion of gender diversity content, reflecting broader societal stigma around gender discourse.

### 4.2. Limitations and Future Directions

This scoping review has some methodological limitations. First, data charting was completed by a single review, which may have introduced bias or inconsistencies in data extraction. Second, the review did not include gray literature such as reports and university theses, meaning relevant school-based programs may have been excluded, thus limiting the comprehensiveness of the findings. Finally, no formal critical appraisal of the included studies was undertaken, which limits our ability to comment on their methodological rigor. It is recommended that future reviews on this topic aim to address these limitations and extend upon the findings of this study by employing a systematic review methodology and applying a risk-of-bias assessment tool.

Despite these limitations, the findings presented by this review highlight some important gaps in the current literature on this topic and highlight important areas for future investigation. For example, this review highlights that there is a growing number of distinct body image prevention programs, with over two dozen having been trialed in Australian schools; however, only a handful have been evaluated across multiple studies. A notable limitation of the current literature is the absence of published evaluations for several widely implemented Australian-based programs, including the Butterfly Foundation’s *BodyKind* and *Body Bright* programs, which are currently being delivered in Australian primary and high schools. The absence of published effectiveness data for these widely implemented programs reflects a broader evidence gap in prevention research, underscoring the need for real-world evaluations. Future research should aim to address this gap in the literature. Additionally, the variable effectiveness and adverse effects of some programs, like mindfulness interventions, highlight the need for developmentally tailored content. Researchers should consider age-appropriate content and delivery formats to ensure that interventions align with the developmental stage and emotional maturity of students at different ages. Furthermore, this review identifies a need for interventions that explicitly enhance protective factors against body dissatisfaction; an important avenue for future exploration

Given the limited demographic reporting revealed in this review, future studies should also adopt more detailed demographic reporting practices. Comprehensive data on students’ cultural, ethnic, and socioeconomic backgrounds are essential to assess the inclusivity and generalizability of findings and to ensure that prevention efforts are equitable and relevant across diverse student populations.

Finally, this review identified a notable absence of qualitative and mixed-methods research. The lack of studies exploring the lived, phenomenological experiences of young people engaging with prevention programs represents a missed opportunity to acquire a deeper understanding of how and why interventions succeed or fail. As Ciao et al. [62] argue, “we need to make space for multiple forms of quantitative and qualitative knowledge in our conception of what is ‘evidence-based.’” Incorporating diverse methodologies could enrich the evidence base and ensure that programs are not only effective but also meaningful and contextually relevant to participants.

## 5. Conclusions

This scoping review provides a comprehensive synthesis of school-based ED prevention programs implemented in Australia since 2010. The findings highlight a range of program approaches trialed, including cognitive dissonance, media literacy, multicomponent risk factor reduction, mindfulness, self-esteem enhancement, and healthy lifestyle promotion. The prevention programs reviewed varied in design, delivery, and target populations, with most focusing on students in Grades 7–8. Our review found that universal media literacy programs like *Media Smart* showed promising outcomes for boys and girls, while several selective programs demonstrated improvements in body image for girls. While a range of ED prevention programs have been trialed in Australian schools, our review identified that few have been rigorously evaluated or demonstrated sustained effectiveness. Our findings highlight the need for future research to investigate developmentally appropriate, gender-sensitive, and culturally inclusive prevention efforts in schools.

## Figures and Tables

**Table 1:** Characteristics of Included Studies.

Study	Aim and Design	*n*	School Setting, Grades, and Location	Age Mean (SD) or Range; % Girls; Ethnicity	Method of Delivery
Atkinson et al. (2016) [22]	Assess the feasibility, acceptability, and efficacy of a mindfulness-based ED prevention program for adolescent females, delivered by facilitators of high and low training Cluster RCT	347 1. Mindfulness-based (*n* = 136) 2. Dissonance-based (*n* = 108) 3. Control (*n* = 101)	Four high schools (2 catholic, 2 private), Grades 10–12, South Australia	Age: 15.70 (0.77) Gender: 100% girls Caucasian (84%)	Facilitator (experienced and less experienced)
Damiano et al. (2018) [23]	To assess the efficacy and feasibility of a teacher-delivered body image intervention for children aged 5–8 Quasi-experimental	51	Two primary schools (government co-ed), Grades K–2, Victoria	Age: 6.61 (0.60) Gender: 49% girls Ethnicity NR, 64% born in AUS	Teacher
Dunstan et al. (2017) [24]	To assess the efficacy of the HBM Co-ed program for Grade 7 girls and compare the outcomes for girls in single-sex versus co-ed classes. Cluster RCT	200 1. HBM Co-ed class (*n* = 73) 2. HBMe Single-sex class (*n* = 74) 3. Control (*n* = 53)	Five secular state high schools, Grade 7, Victoria	HBM Co-ed group: 12.67 (0.44) Gender: HBM single-sex group: 12.66 (0.38) Control: 12.77 (0.41) Ethnicity NR, 90% born in AUS	Facilitator (psychology research assistant)
Forbes et al. (2023) Study 1 [25]	To evaluate the efficacy and acceptability of the co-educational, teacher-led “Dove Confident Me” program for Australian school girls. Quasi-experimental	432 1. DCM (*n* = 198) 2. Control (*n* = 208)	Private girls’ high school, Grade 8, Queensland	Age: 13.3 (0.49) Gender: 100% girls Ethnicity NR, 78.3% born in AUS	Teacher
Forbes et al. (2023) Study 2 [25]	To examine the efficacy and acceptability of the DCM program with modifications for the Australian audience. Quasi-experimental	596 1. DCM modified (*n* = 242) 2. Control (*n* = 354)	Private girls’ high school, Grade 8, Queensland	Age: 12.8 (0.39) Gender: 100% girls	Teacher
Gordon et al. (2021) [26]	To evaluate the efficacy of “SoMe” in improving body image by reducing internalization of appearance ideals and appearance comparison. Cluster RCT	892 1. SoMe (*n* = 483) 2. Control (*n* = 409)	Eight high schools (five public and three independent), Grades 7–8, Victoria	Age: 12.77 (0.74) Gender: 49% girls Ethnicity NR, 84.2% born in AUS	Facilitator
Johnson et al. (2016) [27]	To evaluate the effectiveness of a transdiagnostic mindfulness-based intervention in addressing anxiety, depression, and eating disorder risk factors Cluster RCT	308 1. .b Mindfulness in Schools (*n* = 132) 2. Control (*n* = 176)	Four coeducational high schools, one primary school, Grade 7–8, South Australia	Age: 13.63 (0.43) Gender: 47.7% girls Ethnicity, place of birth NR	Facilitator
Johnson et al. (2019) [28]	To assess the feasibility, acceptability, and efficacy of the Mindfulness Training for Teens curriculum in Australian schools. Quasi-experimental	143 1. Mindfulness Training for Teens (*n* = 71) 2. Control (*n* = 75)	Two secondary schools (1 private, 1 public), Grades 8 and 10, South Australia	Year 8 (age = 13.47; SD = 0.35) Year 10 (age = 15.47; SD = 0.4) Gender: 45.9% girls Ethnicity, place of birth NR	Facilitator
Johnson et al. (2021) [29]	To evaluate the efficacy of a mindfulness intervention in reducing transdiagnostic risk factors, including eating disorders Cluster RCT	434 1. Mindfulness Training for Teens (*n* = 217) 2. Control (*n* = 217)	Three secondary schools (2 private, 1 public), Grades 8 and 10, South Australia	Year 8 (age = 13.67; SD = 0.42) Year 10 (age = 15.52; SD = 0.37) Gender: 48.2% girls Ethnicity, place of birth NR	Facilitator
Kristoffersen et al. (2022) [30]	To evaluate the acceptability and feasibility of the video-based cognitive-dissonance intervention RCT	101 1. Cognitive dissonance (*n* = 46) 2. Self-compassion (*n* = 55)	One private, coeducational high school, Grades 10–11, South Australia	Age: 15.80 (0.68) Gender: 50.5% girls Ethnicity, place of birth NR	Video-based
McCabe et al. (2010) [31]	To examine a body image program designed for adolescent boys RCT	421 1. Self-esteem and Healthy Body Image Program (*n* = 203) 2. Control (*n* = 218)	Five high schools, Grades 7–8, Victoria	Intervention: 12.96 (0.78) Control: 13.18 (0.95) Gender: 0% girls Ethnicity, place of birth NR	Facilitator
McCabe et al. (2017) [32]	To examine the effectiveness of a body image program tailored to the gender-specific concerns of young boys and girls RCT	652 1. Healthy Me boys (*n* = 172) girls (*n* = 163) 2. Control boys (*n* = 149) girls (*n* = 168)	Primary school, Grades 3–4, Victoria	Intervention: 8.80 (0.69) Gender: 49% Control: 8.77 (0.72) Gender: 53% girls Ethnicity, place of birth NR	Facilitator
McLean et al. (2017) [33]	To evaluate the effectiveness of a social media literacy program for adolescent girls. Quasi-experimental (pilot)	101 1. Boost Body Confidence and Social Media Savvy (*n* = 64) 2. Control (*n* = 37)	Two high schools (one public, one private), Grade NR, Victoria	Age: 13.13 (0.33) Gender: 100% girls Ethnicity, place of birth NR	Facilitator
McLean et al. (2019) [34]	To isolate the effects of media literacy and appearance comparison content in a body dissatisfaction prevention program for adolescent girls. RCT	260 1. HBM-Media (*n* = 100) 2. HBM-Appearance comparison (*n* = 82) 3. HBM-Eating (*n* = 78)	Four independent high schools, Grade NR, Victoria	Age: 13.09 (0.45) Gender: 100% girls Ethnicity, place of birth NR	Facilitator (postgraduate psychology student and teacher, qualified research assistant)
Richardson & Paxton (2010) [35]	To evaluate the efficacy of the Happy Being Me intervention in reducing body dissatisfaction in young adolescent girls Quasi-experimental	197 1. HBMe (*n* = 104) 2. Control (*n* = 90)	Two Catholic high schools, Grade 7, Victoria	Age: 12.33 (0.34) Gender: 100% girls Ethnicity NR, 89.7% born in Australia	Facilitator
Ross et al. (2013) [36]	To evaluate the efficacy of selective intervention on body satisfaction. Quasi-experimental	60 1. Y’s girl (*n* = 37) 2. Control (*n* = 23)	Five co-educational primary schools, Grade 6, Victoria	Age range: 11–12 Gender: 100% girls Ethnicity NR, 90% born in Australia	Facilitator
Wilksch & Wade (2013) [37]	To assess student enjoyment and perceived value of Life Smart (acceptability), and efficacy RCT	144 1. Life Smart (*n* = 50) 2. Control (*n* = 64)	Independent private high school, Grade 7, South Australia	Age: 12.71 (0.41) Gender: 45% girls Ethnicity, place of birth NR	Facilitator (psychologist)
Wilksch (2015) [38]	To assess the efficacy and acceptability of a universal, teacher-delivered media literacy intervention Quasi-experimental (pilot)	51 1. Media Smart (*n* = 27) 2. Control (*n* = 24)	One high school, Grade 7, Victoria	Age: 12.43 (0.61) Gender: 52.9% girls Ethnicity, place of birth NR	Teacher
Wilksch et al. (2015) [39]	To investigate the efficacy of an obesity-prevention program (Life Smart) and two eating disorder-prevention programs (Media Smart and HELPP) against each other and a no-intervention control condition with young adolescent girls and boys from pre- to post-intervention and over a 12-month follow-up RCT	1316 1. Media Smart (*n* = 269) 2. Life Smart (*n* = 347) 3. HELPP (*n* = 225) 4. Control (*n* = 473)	12 high schools public (*n* = 3), private (*n* = 4) and Catholic (*n* = 5), Grades 7–8, South Australia, Victoria, and Western Australia.	Age: 13.21 (0.68) Gender: 63.8% girls Ethnicity, place of birth NR	Facilitator (postgraduate psychology students)
Wilksch et al. (2017) [40]	To investigate if students’ baseline level of shape and weight concern moderated intervention outcomes RCT—Secondary analysis	Same as above	Same as above	Same as above	Same as above
Wade et al. (2017) [41]	To investigate whether changes in media internalization moderate changes in shape and weight concern RCT—Secondary analysis	616 Media Smart (*n* = 269) Life smart (*n* = 347)	Same as above	Age: 13.02 (0.63) Gender: 67% Ethnicity, place of birth NR	Same as above
Yager et al. (2019) [42]	To determine the efficacy of the ATLAS program on body image related outcomes in an Australian population of adolescent boys. Quasi-experimental	211 1. ATLAS (*n* = 119) 2. Control (*n* = 92)	Single-sex Catholic high school, Grade 10, Victoria	ATLAS: 15.96 (0.40) Control: 15.74 (0.38) Gender: 0% girls ATLAS 64.7% Caucasian; Control 62.0% Caucasian	PE teacher with peer facilitation (student “team leaders”)
Yager et al. (2023) [43]	To examine the efficacy of ‘Goodform’ relative to a waitlist control condition for improving body image, reducing supplement use, and reducing favorable attitudes towards AAS. RCT	488 1. Goodform (*n* = 244) 2. Control (*n* = 244)	Two independent single-sex, three independent co-ed, one public single-sex, three public co-ed high schools, Grades 9–10, New South Wales, South Australia, Victoria, and Queensland.	Age: 14.81 (0.51) Gender: 0% girls 73.02% Australian, background, place of birth NR	Health and PE teachers

Abbreviations: AAS = anabolic androgenic steroid, ATLAS = Athletes Training and Learning to Avoid Steroids, DCM = Dove Confident Me, ED = eating disorder, HBM = Happy Being Me, HELPP = Helping, Encouraging, Listening and Protecting Peer, NR = not reported, PE = physical education, RCT = randomized controlled trial, AUS = Australian.

**Table 2:** Outcomes of included studies.

Study ID	Level of Prevention	Intervention Details	Intervention Duration/ Follow-Up	Data Collected	Key Findings
Atkinson et al. (2016) [22]	Selective	**Content:** The MBI group practiced mindfulness and acceptance of body image-related thoughts, feelings, and images. Adapted from ‘The Mindfulness Mode’. DBI group drawn from ‘The Body Project’, targeting thin-ideal internalization using discussions, roleplays, videos, and written exercises **Control:** Class as usual	3 × 1 weekly sessions Post-intervention, 1 month, 6 months	▪ Weight and shape concerns, and eating disorder symptoms (EDE-Q) ▪ Negative affect (PANAS-X) ▪ Dietary restraint (DEBQ) ▪ Thin-ideal internalization and sociocultural pressures (SATAQ-3) ▪ Psychosocial impairment (CIA) ▪ Mindful acceptance and awareness (CAMM) ▪ Program acceptability (students and teachers, 5-item scale)	No difference was found *between* interventions for expert or non-expert delivered groups. For expert-delivered MBI, students reported greater reductions in weight and shape concern, dietary restraint, eating disorder symptoms, and psychosocial impairment compared to the control after 6 months. Reductions in sociocultural pressures relative to control for expert-delivered DBI. Mindfulness participants reported a lower understanding of concepts, facilitator confidence, and likelihood of continued use compared to dissonance participants. Students and teachers agreed that the content was less novel or relevant for older students.
Damiano et al. (2018) [23]	Universal	**Content:** Achieving Body Confidence for Young Children (ABC-4-YC) activity and discussion-based lessons centered around a children’s book promoting body acceptance and body diversity **Control**: Nil	3 × 1 h sessions No follow-up	Children: ▪ Body esteem (BES) ▪ Weight stigma (figure rating scale) ▪ Thin-ideal internalization (SATAQ-3) ▪ Appearance-based peer teasing (POTS) Teachers: ▪ Acceptability and feasibility feedback questionnaire	Significant post-intervention increases in body esteem scores. No significant differences in the internalization of appearance ideals, frequency of appearance-based teasing, or weight stigma. Positive teacher feedback for perceived value of activities, student engagement, and practicality
Dunstan et al. (2017) [24]	Selective (girls-only class) Universal (co-ed class)	**Content:** HBM Co-ed features group and individual activities addressing internalization of the thin media ideal, appearance comparisons, low self-esteem, weight-related teasing, and appearance conversations **Control:** No intervention control group	6 × 1 weekly sessions 6-month	▪ Body dissatisfaction (EDI) ▪ Thin-ideal internalization (SATAQ-3) ▪ Appearance comparisons (PACS) ▪ Appearance conversations (ACS) ▪ Dietary restraint (DEBQ) ▪ Self-esteem (RSES) ▪ Weight-related teasing (MRFS-peers)	No significant differences *between* single-sex and co-educational delivery groups for adolescent girls—girls in both groups showed significant improvements over time in body dissatisfaction, internalization, appearance comparisons, self-esteem, appearance conversations, and dietary restraint. Significant post-intervention improvements in body dissatisfaction, thin-ideal internalisation, appearance comparisons, and self-esteem. All but body satisfaction was maintained at 6-month follow-up for HBM Co-ed compared to control.
Forbes et al. (2023) Study 1 [25]	Selective	**Content:** DCM sessions address media literacy, appearance ideals, social comparisons, and boosting self-esteem through class discussions, videos, group activities, and written exercises. **Control**: class as usual (wellbeing lessons)	4 × 1 fortnightly sessions 3-month	▪ Body esteem (BESAA) ▪ Body appreciation BAS ▪ Thin-ideal internalization (SATAQ-3) ▪ Sociocultural pressures (PSPS) ▪ Social comparisons (SCMPS) ▪ Appearance-related teasing (EAT-III) ▪ Appearance conversations (ACS) ▪ Negative affect ▪ Self-esteem (RSES-S) ▪ Dietary restraint (DEBQ) ▪ Life engagement and future plans ▪ Program acceptability (student questionnaire, 10-item teacher evaluation survey,) ▪ Content fidelity (self-report)	Students in the DCM group reported worse levels of sociocultural pressure post-test compared to the control group. The control group also showed greater reduction in social comparisons compared to the DCM group. No other significant changes across outcomes at post-intervention or 3-month follow-up High student acceptability ratings for comfort and teacher effectiveness, moderate acceptability for importance, and low-moderate acceptability for enjoyment and helpfulness of the content. Teacher feedback indicated low student engagement and perceived efficacy. Content implementation fidelity rated between 65 and 100% across 4 sessions.
Forbes et al. (2023) Study 2 [25]	Selective	**Content:** Modified DCM program replaced worksheets with class discussions, including videos featuring Australian adolescents and added a new discussion topic ‘Conversations on Instagram’. **Control**: class as usual (wellbeing lessons)	4 × 1 fortnightly sessions 3-month	Same as above, added: ▪ Ideal Body Stereotype Scale—Revised (IBSS-R), ▪ Maternal Pressure Scale Removed: ▪ EAT-III TS, negative affect, life engagement, and future plans scales	Significantly greater thin-ideal internalization was reported at post-test and maintained at 3-month follow-up for the intervention group compared to the control. Significantly higher sociocultural pressure at post-test compared to the comparison group. No significant differences were found between groups for self-esteem, body esteem, body appreciation, social comparison, appearance talk, maternal pressure, and dietary restraint from pre-test to post-test or 3-month follow-up. Students rated the modified DCM program significantly higher for enjoyment, helpfulness, teacher effectiveness, and importance compared to the original program.
Gordon et al. (2021) [26]	Universal	Intervention: **“SoMe**” social media literacy program that teaches students to critically analyze social media content, including ads, celebrity posts, and friends’ pages, consider realism, respond to negative feedback, use social media for positive change, and present their ‘real’ selves with less focus on appearance. **Control**: class as usual	4 × 50 min weekly sessions 6- and 12-month	Primary: ▪ Weight and shape concern (EDE-Q) ▪ Body dissatisfaction (VAS) ▪ Dietary restraint (DEBQ) ▪ Strategies to Increase Muscles (BDI) Secondary: ▪ Depression (CESDR-10) ▪ Self-esteem (RSES single item) ▪ Exploratory: ▪ Appearance-ideal internalization (SATAQ-3 adapted) ▪ Muscular ideal internalization (SATAQ-4) ▪ Upward appearance comparisons (UPACS)	Whole sample: SoMe did not produce significant changes across time and outcomes compared to control. Girls: - The intervention group exhibited reduced dietary restraint from baseline to the 6-month follow-up. Weaker increase in depressive symptoms from baseline to the 6-month follow-up compared to the control group Boys: - The intervention group showed an increase in self-esteem from baseline to the 6-month follow-up and an increase in drive for muscularity from baseline to the 12-month follow-up. Weak reduction in drive to increase muscularity from baseline to 12-month follow-up.
Johnson et al. (2016) [27]	Universal	**Intervention:** .b (“Dot be”) teaches mindfulness concepts such as attention training, and experiential practices such as body scan, relaxation techniques, mindfulness of breathing **Control**: class as usual	8 × 35–60 min weekly sessions 3--month	▪ Weight and shape concern (EDE-Q) ▪ Anxiety and depression (DASS-21) ▪ Psychological wellbeing (WEMWBS) ▪ Mindfulness (CAMM) ▪ Difficulties in Emotion Regulation (DERS) ▪ Self-compassion (SCS) ▪ Student and teacher acceptability questionnaires	No main effects of group or group-time interactions for any primary or secondary outcome variable. Homework completion did not moderate effects. At 3-month follow-up, males in the intervention group, as well as those with low baseline weight/shape and depression, exhibited higher anxiety levels compared to the control group. High acceptability of the program amongst students and teachers
Johnson et al. (2019) [28]	Universal	**Intervention:** Mindfulness Training for Teens classes were conducted in rooms separate from the actual classroom. Sessions included 10–20 min of meditation, classroom discussions, and presentation of mindfulness concepts. Students received a weekly handout summarizing the lesson and instructions for formal and informal home practices and meditation audio files. **Control**: class as usual	8 × 90 min weekly sessions 4-month	▪ Weight and shape concern (EDE-Q) ▪ Generalized anxiety symptoms (GAD-7) ▪ Depression (DASS-21) ▪ Psychological wellbeing (WEMWBS) ▪ Feasibility of delivering the program in schools ▪ Staff acceptability, ▪ Student acceptability surveys ▪ Fidelity ratings	Psychological outcomes: - No significant differences at post-intervention between groups - At the 4-month follow-up, the intervention group showed moderate improvements in depression and anxiety compared to the control group. - Moderator analyses revealed these results were confined to the Year 10 students. Feasibility: - A total of 3/5 schools declined to participate due to the inability to accommodate sessions - Participating schools were able to provide a school counselor to attend sessions Acceptability: - Students rated the content as acceptable in terms of enjoyment, learning - Post-intervention, 27.1% of students completed home practice weekly, dropping to ≤ 8.0% at four-month follow-up. Fidelity and competence: - Lesson content fidelity rated as proficient by the program developer - Students rated instructor competence 8.9/10
Johnson et al. (2021) [29]	Universal	**Intervention:** Mindfulness Training for Teens (as per pilot study) with slightly shorter lessons with the removal of a 10 min break **Control**: class as usual	8 × 65–75 min weekly sessions 9-month follow-up (*n* = 161)	▪ Weight and shape concern (EDE-Q) ▪ Mindfulness (CHIME-A) ▪ Generalized anxiety symptoms (GAD-7) ▪ Depression (DASS-21) ▪ Psychological wellbeing (WEMWBS) ▪ Student course acceptability survey ▪ Staff course acceptability ratings	Post-intervention, there were no significant differences between the mindfulness and control groups on any psychological outcomes. Staff perceived student enjoyment, interest, and learning as high, on average. The mean course ratings from students were: enjoyment and interest 5.61, amount learnt 5.98, and likelihood of using mindfulness practices in the future 5.30. At the 3-month follow-up, the mindfulness group had a slight decrease in ‘Decentering and Nonreactivity’, with no other psychological differences. Year 8 students showed no improvement post-intervention and exhibited worse mindfulness and wellbeing at the 3-month follow-up. Year 10 students showed no significant differences at any time point.
Kristoffersen et al. (2022) [30]	Universal	**Intervention:** Cognitive-dissonance intervention—15 min video highlighting the costs of pursuing media appearance ideals and practices challenging these ideals. **Control:** Self-compassion intervention—15 min video focused on the costs of being self-critical and practice being self-compassionate with home worksheet.	1 × 15 min video N/A	▪ ED risk factors (VAS) ▪ Weight and shape concern (EDE-Q) ▪ Body Appreciation (BAS-2C) ▪ Thin-ideal internalization (SATAQ-4) ▪ Muscular ideal internalization (SATAQ-4) ▪ Sociocultural pressure (SATAQ-4) ▪ Positive and negative affect (PANAS-C) ▪ Self-compassion (SCS) ▪ Self-criticism (FSCRS-S) ▪ Student acceptability ratings	Reductions across state outcomes were larger for girls in the SC group compared to CD, and vice versa for boys. Positive mood decreased substantially for boys in the CD condition. Acceptability: - Self-compassion intervention demonstrated higher acceptability scores for both genders across all outcomes. Irrelevance of content was endorsed more commonly among boys. Students liked the content themes, relevance, video format, use of “real world” examples, and ability to complete in-class worksheets at their own pace. At follow-up, more girls (60.9%) than boys (16.7%) reported using the self-compassion techniques during the week. Conversely, more boys (26.7%) used the CD techniques than girls (4.5%). The most common responses for non-engagement cited lack of interest, relevance, time, or forgetting.
McCabe et al. (2010) [31]	Universal (boys only)	**Intervention:** Self-Esteem and Body Image Program sessions focused on valuing personality over appearance, reducing negative comparisons, building communication and social skills, developing coping strategies for stress, and consolidating learning through revision activities. **Control**: students completed questionnaires at 5 time points	5 × 60 min sessions 3-month, 6-month, 12-month	▪ Drive for thinness (EDI) ▪ Negative affect (DASS-21) ▪ Self-esteem (SDQ-II) ▪ Media influences (PSI-BIBCQ) ▪ Same-sex and opposite-sex peer popularity (SDQ-II)	No significant differences between the intervention and control group across all nine outcomes, at any time point. Secondary analysis revealed that boys with higher body image concerns experienced less negative affect compared to control.
McCabe et al. (2017) [32]	Universal	**Intervention:** Healthy Me sessions aimed to promote acceptance of diversity, positive self-image, and healthy peer relationships. Gender-specific activities supported boys in valuing diverse talents and teamwork, while girls focused on body acceptance and coping with appearance-related pressures. Homework tasks involved family participation to reinforce learning at home.	5 × 60 min sessions 3-month	Primary: ▪ Body esteem (BES) ▪ Muscle esteem (purpose built) ▪ Body change strategies (BCI-A) Secondary: ▪ Sociocultural influences on body image ▪ Investment in athletic/muscular appearance (MAPI) ▪ Fruit and vegetable intake	Increases in body esteem were observed for Healthy Me boys and girls at 3-month follow-up, while muscle esteem improved post-intervention and was maintained at follow-up, compared to control. Boys in the intervention group showed a significant decrease in investment in masculine gender norms at both post-intervention and recap compared to the control group.
McLean et al. (2017) [33]	Selective	**Intervention:** Boost Body Confidence and Social Media Savvy lessons focus on enhancing students’ critical engagement with social media and reducing appearance comparisons and comments. Based on the Happy Being Me program. **Control**: class as usual	3 × 50 min sessions N/A	Weight and shape concern (EDE-Q) Thin-ideal internalization (SATAQ-4) Body esteem (BES) Dietary restraint (DEBQ) Upward appearance comparison (UPACS) Appearance conversations (ACS) Fear of fat Fear of negative appearance evaluation (FNAES) Realism skepticism Critical thinking	Students who received ‘Boost’ reported significant improvements in body esteem, dietary restraint, and realism skepticism compared to students in the control group.
McLean et al. (2019) [34]	Selective	**Interventions:** Happy Being Me—Media sessions focused on identifying unrealistic beauty ideals in media, understanding manipulation techniques, and challenging perceived gains of conforming to beauty ideals. Happy Being Me—Appearance comparison sessions helped students recognize the harms of upward comparisons, understand the role of peer influence, and develop strategies to resist comparison pressures. Happy Being Me—Healthy eating sessions distinguished between dieting and healthy eating, emphasized internal cues like hunger and fullness, discouraged dieting, and addressed harmful food-related language.	3 sessions 3-month	▪ Body dissatisfaction (EDI) ▪ Bulimic symptoms (EDI) ▪ Thin-ideal internalization (SATAQ-3) ▪ Dietary restraint (DEBQ) ▪ Appearance comparisons (PACS, UPACS, DPACS) ▪ Appearance conversations (ACS) ▪ Media literacy (realism skepticism, Critical Thinking About Media Messages, Critical Thinking about Media Messages—Appearance Focus) ▪ Fear of negative appearance evaluation (FNAES) ▪ Content fidelity	No significant differences were found between groups for any outcome variable. High-risk participants showed reductions in body dissatisfaction sustained through follow-up and short-term improvements in bulimic symptoms and upward appearance comparison after HBM-Comparison. At follow-up, further gains were observed in thin-ideal internalization, appearance comparison, and fear of negative evaluation. High-risk girls in the HBM–Media group also showed improvements in appearance comparison (post and follow-up), though critical thinking about media declined post-program.
Richardson & Paxton (2010) [35]	Selective	**Intervention:** Happy Being Me seeks to inform participants about the impacts of thin-ideal internalization, engaging in body comparisons, discussing appearances, and experiencing appearance-related teasing. Content is delivered through presentations, worksheets, role plays, discussions, and videos. **Control:** class as usual	3 × 50 min sessions 3-month	▪ Body dissatisfaction (EDI) ▪ Body satisfaction (VAS) ▪ Bulimic symptoms (EDI) ▪ Thin-ideal internalization ▪ Dietary restraint (DEBQ) ▪ Body comparison (PACS) ▪ Appearance conversations (ACS) ▪ Appearance-based peer teasing (POTS) ▪ Self-esteem (RSES) ▪ Students’ knowledge of intervention topics ▪ Quantitative and qualitative feedback (optional)	Girls who received HBM showed significant improvements in knowledge of intervention topics, thin-ideal internalization, body comparisons, appearance-related conversations with peers, body satisfaction, dietary restraint, and self-esteem compared to control. Majority of girls rated HBM as interesting and enjoyable.
Ross et al. (2013) [36]	Selective	**Intervention:** Y’s Girl sessions utilized group discussions, reflective exercises, and roleplays to promote body confidence, self-esteem, and communication skills. Sessions involved exploring friendship values, cultural beauty practices, body positivity, media literacy, and assertiveness, while encouraging positive self-talk and resilience.	6 × 60 min sessions N/A	▪ Body esteem (BES) ▪ Ideal-perceived body discrepancy (CDRS) ▪ Thin-ideal internalization (SATAQ-3) ▪ Quality of peer relationships (IPPA) ▪ Body comparisons (PACS) ▪ Appearance conversations (ACS) ▪ Bulimic symptoms (EDI) ▪ Self-esteem (RSES)	Significant improvements in body satisfaction, body size discrepancy, thin-ideal internalization, body comparisons, and self-esteem compared to control participants. Changes in body image were moderated by baseline levels of self-esteem and appearance-based conversations with peers.
Wilksch & Wade (2013) [37]	Universal	**Intervention:** Media Smart lessons cover topics such as awareness of culturally promoted body ideals, techniques used by the media to promote body ideals, and encouraging students to engage in body activism. **Control**: class as usual	8 × 50 min sessions N/A	▪ Shape and weight concern (EDE-Q) ▪ Dietary restraint (DEBQ) ▪ Body dissatisfaction (EDI) ▪ Thin-ideal internalization (SATAQ-3) ▪ Depression (CDI-SF) ▪ Concern over mistakes (FMPS) ▪ Weight-related teasing (MRFS-peers) ▪ Regular eating (Project EAT) ▪ Screen time (GUTS) ▪ Physical activity (GUTS) ▪ Program feedback: Student self-report on enjoyment, value, learning, and recommendations for improvement	Girls in the Life Smart group showed significant improvement in shape and weight concern at post-intervention compared to controls. Life Smart boys showed no significant changes compared to the control, aside from an increase in physical activity.
Wilksch (2015) [38]	Universal	**Intervention:** Media Smart lessons cover topics such as awareness of culturally promoted body ideals, techniques used by the media to promote body ideals, and encouraging students to engage in body activism. **Control**: class as usual	8 × 50 min sessions 6-month	▪ Shape and weight concern (EDE-Q) ▪ Dietary restraint (DEBQ) ▪ Media Internalization (SATAQ-3) ▪ Feelings of ineffectiveness (EDI) ▪ Self-esteem (RSES) ▪ Depression (CDI-SF) ▪ Weight-related teasing (MRFS-peers) ▪ Teacher report: Content fidelity, perceived value for students, qualitative feedback.	Media Smart girls scored significantly lower than control peers on feelings of ineffectiveness and weight-related peer teasing at post-program, with the latter also true at 6-month follow-up. Boys showed the most significant improvements in reducing feelings of ineffectiveness and weight-related peer teasing at 6 -month follow-up. Nonsignificant improvement in shape and weight concern at post-intervention.
Wilksch et al. (2015) [39]	Universal	**Interventions:** Groups involved interactive components such as group discussions, presentations, videos, and role plays. 1. Media Smart—targets media internalization, described above. 2. Life Smart—healthy lifestyle promotion and obesity prevention 3. HELPP—based on Happy Being Me, targeting internalization of social appearance ideals and appearance comparisons. **Control**: class as usual	8 × 50 min sessions 6- and 12-month	▪ Shape, weight, and eating concern (EDE-Q) ▪ Thin-ideal internalization (SATAQ-3) ▪ Dietary restraint (DEBQ) ▪ Body dissatisfaction (EDI) ▪ Depression (CDI-SF) ▪ Weight-related teasing (MRFS-peers) ▪ Concern over mistakes (FMPS) ▪ Sociocultural pressures (PSPS) ▪ Regular eating (Project EAT) ▪ Screen time (GUTS) ▪ Physical activity (GUTS)	Girls in the Media Smart and HELPP programs exhibited reductions in weight and shape concern compared to Life Smart girls at 12 months. Media Smart girls had fewer eating concerns than HELPP girls at 6 months. Boys in the Media Smart program showed lower media internalization, maintained at 12-month follow-up compared to control boys. Perceived Pressure: Media Smart and control girls scored lower than HELPP girls at 6 months. Media Smart Boys: Significant benefits at post-program for body dissatisfaction, media internalization, weight-related peer teasing, and perfectionism; benefits at 6 and 12 months for media internalization and depression. Life Smart Boys: Only benefit was on body dissatisfaction post-program; higher media internalization at post-program and 6-month; higher depression at 6 and 12 months. HELPP Boys: Benefits on media internalization at post-program and 6 months; benefits on depression at 6 months; higher weight-related peer teasing at post-program. Overall: The Media Smart group had lower mean scores across all follow-up points than the control group. No significant differences were found for BMI.
Wilksch et al. (2017) [40]	Same as above				For students with higher baseline shape and weight concerns, Media Smart led to reductions in shape, weight, and eating concerns after Media Smart was maintained at 12 months, and less meal skipping at 12 months compared to HELPP. For students with lower baseline shape and weight concerns, Media Smart and Life Smart reduced body dissatisfaction post-program.
Wade et al. (2017) [41]	Same as above	**Intervention:** Media Smart and Life Smart	Same as above	▪ Thin-ideal internalization (SATAQ-3) ▪ Shape, weight, and eating concern (EDE-Q)	Media Smart was associated with significantly lower media internalization post-intervention. Higher media internalization levels were significantly associated with greater increases in weight and shape concerns at 12 months. Media internalization accounted for 48% of the variance for weight concerns and 33% of the variance for shape concerns.
Yager et al. (2019) [42]	Universal	**Intervention:** Athletes Training and Learning to Avoid Steroids (ATLAS) sessions focus on drug and supplement education, strength training, and sports nutrition. **Control:** Waitlist control	10 × 45 min sessions 3-month	▪ Weight and appearance esteem (BESAA) ▪ Drive for Muscularity (DMS) ▪ Body Appearance Ratings ▪ Functional body satisfaction (EIS) ▪ Self-report knowledge about supplements, attitudes toward supplement use, intentions to use substances, use of alcohol, use of steroids, protein powder, and creatine ▪ Program feasibility: student and teacher feedback	Non-significant improvements in functional and esthetic body image satisfaction for ATLAS participants. A non-significant increase in negative attitudes toward the use of appearance- and performance-enhancing drugs was found and maintained at 3-month follow-up, compared to the control. Feedback from teachers included condensing the program to 5–6 sessions, incorporating audio–visual material, and enhancing the clarity of the manual.
Yager et al. (2023) [43]	Universal	**Intervention:** Goodform sessions aim to improve body image, reduce positive outcome expectations for anabolic androgenic steroid use, and reduce intentions to use muscle-enhancing supplements in mid-adolescent boys. Content was adapted from ‘The Body Project: More than Muscles’ and the ATLAS program. **Control:** Waitlist control	4 × 45–60 min sessions 2-month	▪ Body satisfaction (MBAS-R) ▪ Thin and muscular-ideal internalization (SATAQ-4R) ▪ Sociocultural pressures (SATAQ-4R) ▪ Negative body talk (MBTS) ▪ Attitudes towards AAS (O-AAS) ▪ Intentions to use AAS (I-ASS) ▪ Actual use of APES. ▪ Social norms for AAS and supplement use ▪ Teacher feedback on adherence to lessons, student engagement/enjoyment, and understanding of the content.	No improvements in body image, supplement use, or intentions to use anabolic steroids compared to the control group. Boys’ muscularity dissatisfaction, appearance pressures, and attitudes towards using AAS all increased over time, regardless of condition. On average, 70.31% of topics were fully completed, 26.56% of topics were partially completed, and 3.13% of topics were not covered at all

Abbreviations: ACS, Appearance Conversations Scale; BAS, Body Appreciation Scale; BES, Body Esteem Scale for Children; CAMM, Child and Adolescent Mindfulness Measure; CDI, Child Depression Inventory; CDRS, Contour Drawing Rating Scale; CESDR-10, Centre for Epidemiological Studies Depression Scale Revised; CHIME-A, Comprehensive Inventory of Mindfulness Experiences—Adolescents; CIA, Clinical Impairment Assessment; DASS-21, Depression Anxiety Stress Scales; DEBQ, Dutch Eating Behavior Questionnaire; DERS, Difficulties in Emotion Regulation Scale; DMS, Drive for Muscularity Scale; EDE-Q, eating disorder questionnaire; EDI, Eating Disorder Inventory; EIS, Embodied Image Scale; FMPS, Frost Multidimensional Perfectionism Scale; FSCRS-S, Forms of Self-Criticizing/Attacking and Self-Reassuring Scale–Short; FNAES, Fear of Negative Appearance Evaluation Scale; GUTS, Growing Up Today Study; I-AAS/O-AAS, Attitudes Toward Anabolic-Androgenic Steroids; IPPA, Inventory of Parent and Peer Attachment; MBTS, Male Body Talk Scale; MAPI, Muscularity and Athleticism Perception Inventory; MBAS-R, Male Body Attitudes Scale–Revised; MRFS, McKnight Risk Factor Survey; PACS, Physical Appearance Comparison Scale; PANAS-X, Positive and Negative Affect Schedule-Expanded; POTS, Perception of Teasing Scale; PSI-BIBCQ, Peer and Social Influences–Body Image and Body Change Questionnaire; PSPS, Perceived Sociocultural Pressure Scale; SATAQ, Sociocultural Attitudes Towards Appearance Scale; SCS, Self-Compassion Scale; SCMPS, Social Comparison and Media Pressure Scale; SDQ-II, Self-Description Questionnaire II; UPACS, Upward Physical Appearance Comparison Scale; VAS, Visual Analog Scales; WEMWBS, Warwick-Edinburgh Mental Wellbeing Scale.

## Data Availability

No new data were created or analyzed in this study. Data sharing is not applicable to this article.

## Supplementary Materials

The following supporting information can be downloaded at: https://www.mdpi.com/article/10.3390/nu17132118/s1, PRISMA Checklist included as a Supplementary File.

## Appendix A

APA PsycINFO Search Strategy

1. eating disorder*.mp. or exp Eating Disorders/
2. binge eating.mp. or exp binge eating/
3. muscle dysmorphi*.mp. or exp muscle dysmorphia/
4. (other specified feeding and eating).mp.
5. emotional eating.mp. or exp emotional eating/
6. exercise dependence.mp. or exp exercise dependence/
7. body image.mp. or Body Image/
8. 1 or 2 or 3 or 4 or 5 or 6 or 7
9. early intervention.mp. or exp Early Intervention/
10. prevent*.mp. or exp Prevention/
11. universal prevention.mp.
12. selective prevention.mp.
13. 9 or 10 or 11 or 12
14. exp Schools/or school*.mp.
15. 8 and 13 and 14
16. limit 15 to english language

## References

[1] Udo T., Grilo C.M. (2019). Psychiatric and Medical Correlates of DSM-5 Eating Disorders in a Nationally Representative Sample of Adults in the United States. Int. J. Eat. Disord..

[2] Hay P., Mitchison D., Collado A.E.L., González-Chica D.A., Stocks N., Touyz S. (2017). Burden and Health-Related Quality of Life of Eating Disorders, Including Avoidant/Restrictive Food Intake Disorder (ARFID), in the Australian Population. J. Eat. Disord..

[3] Miskovic-Wheatley J., Bryant E., Ong S.H., Vatter S., Le A., Aouad P., Barakat S., Boakes R., Brennan L., Bryant E. (2023). Eating Disorder Outcomes: Findings from a Rapid Review of over a Decade of Research. J. Eat. Disord..

[4] Deloitte Access Economics (2024). Paying the Price, Second Edition: The Economic and Social Impact of Eating Disorders in Australia.

[5] National Eating Disorders Collaboration (NEDC) (2023). National Eating Disorders Strategy 2023–2033.

[6] National Eating Disorders Collaboration (NEDC) (2023). Eating Disorders in Schools: Prevention, Early Identification, Response and Recovery Support.

[7] Pastore M., Indrio F., Bali D., Vural M., Giardino I., Pettoello-Mantovani M. (2023). Alarming Increase of Eating Disorders in Children and Adolescents. J. Pediatr..

[8] Sparti C., Santomauro D., Cruwys T., Burgess P., Harris M. (2019). Disordered Eating among Australian Adolescents: Prevalence, Functioning, and Help Received. Int. J. Eat. Disord..

[9] Morris A., Elliott E., Madden S. (2022). Early-Onset Eating Disorders in Australian Children: A National Surveillance Study Showing Increased Incidence. Int. J. Eat. Disord..

[10] Yager Z. (2024). Something, Everything, and Anything More than Nothing: Stories of School-Based Prevention of Body Image Concerns and Eating Disorders in Young People. Eat. Disord..

[11] Yager Z., O’Dea J.A. (2015). School-Based Prevention. The Wiley Handbook of Eating Disorders.

[12] Boat T., O’Connell M.E., Warner K.E., National Research Council, Institute of Medicine (2009). Defining the Scope of Prevention. Preventing Mental, Emotional, and Behavioral Disorders Among Young People: Progress and Possibilities.

[13] Berry S.L., Burton A.L., Rogers K., Lee C.M., Berle D.M. (2025). A Systematic Review and Meta-Analysis of Eating Disorder Preventative Interventions in Schools. Eur. Eat. Disord. Rev..

[14] Pursey K.M., Burrows T.L., Barker D., Hart M., Paxton S.J. (2021). Disordered Eating, Body Image Concerns, and Weight Control Behaviors in Primary School Aged Children: A Systematic Review and Meta-Analysis of Universal–Selective Prevention Interventions. Int. J. Eat. Disord..

[15] Chua J.Y.X., Tam W., Shorey S. (2020). Research Review: Effectiveness of Universal Eating Disorder Prevention Interventions in Improving Body Image among Children: A Systematic Review and Meta-Analysis. J. Child Psychol. Psychiatry.

[16] Wong R.S., Chan B.N.K., Lai S.I., Tung K.T.S. (2024). School-Based Eating Disorder Prevention Programmes and Their Impact on Adolescent Mental Health: Systematic Review. BJPsych Open.

[17] Perrin J.M., Cheng T.L., National Academies of Sciences, Engineering, and Medicine (2024). Opportunities for Health Promotion and Disease Prevention in Schools. Launching Lifelong Health by Improving Health Care for Children, Youth, and Families.

[18] Munn Z., Peters M.D.J., Stern C., Tufanaru C., McArthur A., Aromataris E. (2018). Systematic Review or Scoping Review? Guidance for Authors When Choosing between a Systematic or Scoping Review Approach. BMC Med. Res. Methodol..

[19] Peters M.D.J., Godfrey C., McInerney P., Munn Z., Tricco A.C., Khalil H., Aromataris E., Lockwood C., Porritt K., Pilla B., Jordan Z. (2024). Scoping Reviews. JBI Manual for Evidence Synthesis.

[20] Tricco A.C., Lillie E., Zarin W., O’Brien K.K., Colquhoun H., Levac D., Moher D., Peters M.D.J., Horsley T., Weeks L. (2018). PRISMA Extension for Scoping Reviews (PRISMA-ScR): Checklist and Explanation. Ann. Intern. Med..

[21] Mrazek P.J., Haggerty R.J., Institute of Medicine (US) Committee on Prevention of Mental Disorders (1994). Reducing Risks for Mental Disorders: Frontiers for Preventive Intervention Research.

[22] Atkinson M.J., Wade T.D. (2016). Does Mindfulness Have Potential in Eating Disorders Prevention? A Preliminary Controlled Trial with Young Adult Women. Early Interv. Psychiatry.

[23] Damiano S.R., Yager Z., McLean S.A., Paxton S.J. (2018). Achieving Body Confidence for Young Children: Development and Pilot Study of a Universal Teacher-Led Body Image and Weight Stigma Program for Early Primary School Children. Eat. Disord. J. Treat. Prev..

[24] Dunstan C.J., Paxton S.J., McLean S.A. (2017). An Evaluation of a Body Image Intervention in Adolescent Girls Delivered in Single-Sex versus Co-Educational Classroom Settings. Eat. Behav..

[25] Forbes J., Paxton S., Yager Z. (2023). Independent Pragmatic Replication of the Dove Confident Me Body Image Program in an Australian Girls Independent Secondary School. Body Image.

[26] Gordon C.S., Jarman H.K., Rodgers R.F., McLean S.A., Slater A., Fuller-Tyszkiewicz M., Paxton S.J. (2021). Outcomes of a Cluster Randomized Controlled Trial of the Some Social Media Literacy Program for Improving Body Image-Related Outcomes in Adolescent Boys and Girls. Nutrients.

[27] Johnson C., Burke C., Brinkman S., Wade T. (2016). Effectiveness of a School-Based Mindfulness Program for Transdiagnostic Prevention in Young Adolescents. Behav. Res. Ther..

[28] Johnson C., Wade T. (2019). Piloting a More Intensive 8-week Mindfulness Programme in Early- and Mid-adolescent School Students. Early Interv. Psychiatry.

[29] Johnson C., Wade T. (2021). Acceptability and Effectiveness of an 8-Week Mindfulness Program in Early- and Mid-Adolescent School Students: A Randomised Controlled Trial. Mindfulness.

[30] Kristoffersen M., Johnson C., Atkinson M.J. (2022). Feasibility and Acceptability of Video-Based Microinterventions for Eating Disorder Prevention among Adolescents in Secondary Schools. Int. J. Eat. Disord..

[31] McCabe M.P., Ricciardelli L.A., Karantzas G. (2010). Impact of a Healthy Body Image Program among Adolescent Boys on Body Image, Negative Affect, and Body Change Strategies. Body Image.

[32] McCabe M.P., Connaughton C., Tatangelo G., Mellor D., Busija L. (2017). Healthy Me: A Gender-Specific Program to Address Body Image Concerns and Risk Factors among Preadolescents. Body Image.

[33] McLean S.A., Wertheim E.H., Masters J., Paxton S.J. (2017). A Pilot Evaluation of a Social Media Literacy Intervention to Reduce Risk Factors for Eating Disorders. Int. J. Eat. Disord..

[34] McLean S.A., Wertheim E.H., Marques M.D., Paxton S.J. (2019). Dismantling Prevention: Comparison of Outcomes Following Media Literacy and Appearance Comparison Modules in a Randomised Controlled Trial. J. Health Psychol..

[35] Richardson S.M., Paxton S.J. (2010). An Evaluation of a Body Image Intervention Based on Risk Factors for Body Dissatisfaction: A Controlled Study with Adolescent Girls. Int. J. Eat. Disord..

[36] Ross A., Paxton S.J., Rodgers R.F. (2013). Y’s Girl: Increasing Body Satisfaction among Primary School Girls. Body Image.

[37] Wilksch S.M., Wade T.D. (2013). Life Smart: A Pilot Study of a School-Based Program to Reduce the Risk of Both Eating Disorders and Obesity in Young Adolescent Girls and Boys. J. Pediatr. Psychol..

[38] Wilksch S.M. (2015). School-Based Eating Disorder Prevention: A Pilot Effectiveness Trial of Teacher-Delivered Media Smart. Early Interv. Psychiatry.

[39] Wilksch S.M., Paxton S.J., Byrne S.M., Austin S.B., Thompson K.M., Dorairaj K., Wade T.D. (2015). Prevention Across the Spectrum: A Randomized Controlled Trial of Three Programs to Reduce Risk Factors for Both Eating Disorders and Obesity. Psychol. Med..

[40] Wilksch S.M., Paxton S.J., Byrne S.M., Austin S.B., O’Shea A., Wade T.D. (2017). Outcomes of Three Universal Eating Disorder Risk Reduction Programs by Participants with Higher and Lower Baseline Shape and Weight Concern. Int. J. Eat. Disord..

[41] Wade T.D., Wilksch S.M., Paxton S.J., Byrne S.M., Austin S.B. (2017). Do Universal Media Literacy Programs Have an Effect on Weight and Shape Concern by Influencing Media Internalization?. Int. J. Eat. Disord..

[42] Yager Z., McLean S.A., Li X. (2019). Body Image Outcomes in a Replication of the ATLAS Program in Australia. Psychol. Men Masc..

[43] Yager Z., Doley J.R., McLean S.A., Griffiths S. (2023). Goodform: A Cluster Randomised Controlled Trial of a School-Based Program to Prevent Body Dissatisfaction and Muscle Building Supplement Use Among Adolescent Boys. Body Image.

[44] Fairburn C.G., Beglin S.J. (1994). Assessment of Eating Disorders: Interview or Self-Report Questionnaire?. Int. J. Eat. Disord..

[45] Van Strien T., Frijters J.E.R., Bergers G.P.A., Defares P.B. (1986). The Dutch Eating Behavior Questionnaire (DEBQ) for Assessment of Restrained, Emotional, and External Eating Behavior. Int. J. Eat. Disord..

[46] Thompson J.K., van den Berg P., Roehrig M., Guarda A.S., Heinberg L.J. (2004). The Sociocultural Attitudes towards Appearance Scale-3 (SATAQ-3): Development and Validation. Int. J. Eat. Disord..

[47] Stice E., Marti C.N., Spoor S., Presnell K., Shaw H. (2008). Dissonance and Healthy Weight Eating Disorder Prevention Programs: Long-Term Effects from a Randomized Efficacy Trial. J. Consult. Clin. Psychol..

[48] Wilksch S.M., Tiggemann M., Wade T.D. (2006). Impact of Interactive School-based Media Literacy Lessons for Reducing Internalization of Media Ideals in Young Adolescent Girls and Boys. Int. J. Eat. Disord..

[49] Yager Z., Diedrichs P.C., Ricciardelli L.A., Halliwell E. (2013). What Works in Secondary Schools? A Systematic Review of Classroom-Based Body Image Programs. Body Image.

[50] Stice E., Shaw H., Marti C.N. (2007). A Meta-Analytic Review of Eating Disorder Prevention Programs: Encouraging Findings. Annu. Rev. Clin. Psychol..

[51] Le L.K.-D., Barendregt J.J., Hay P., Mihalopoulos C. (2017). Prevention of Eating Disorders: A Systematic Review and Meta-Analysis. Clin. Psychol. Rev..

[52] Kusina J.R., Exline J.J. (2019). Beyond Body Image: A Systematic Review of Classroom-Based Interventions Targeting Body Image of Adolescents. Adolesc. Res. Rev..

[53] Pursey K.M., Hart M., Hure A., Cheung H.M., Ong L., Burrows T.L., Yager Z. (2022). The Needs of School Professionals for Eating Disorder Prevention in Australian Schools: A Mixed-Methods Survey. Children.

[54] Piran N. (2010). A Feminist Perspective on Risk Factor Research and on the Prevention of Eating Disorders. Eat. Disord..

[55] Piran N. (2015). A Feminist Perspective on the Prevention of Eating Disorders. The Wiley Handbook of Eating Disorders.

[56] Murray S.B., Rieger E., Karlov L., Touyz S.W. (2013). Masculinity and Femininity in the Divergence of Male Body Image Concerns. J. Eat. Disord..

[57] Atkinson M.J., Stock N.M., Alleva J.M., Jankowski G.S., Piran N., Riley S., Calogero R., Clarke A., Rumsey N., Slater A. (2020). Looking to the Future: Priorities for Translating Research to Impact in the Field of Appearance and Body Image. Body Image.

[58] Becker C.B. (2016). Our Critics Might Have Valid Concerns: Reducing Our Propensity to Conflate. Eat. Disord..

[59] Stice E., Onipede Z.A., Marti C.N. (2021). A Meta-Analytic Review of Trials That Tested Whether Eating Disorder Prevention Programs Prevent Eating Disorder Onset. Clin. Psychol. Rev..

[60] Levine M.P., Smolak L. (2021). Protective Factors. The Prevention of Eating Problems and Eating Disorders: Theories, Research, and Applications.

[61] Granfield P., Kemps E., Johnson C., Seekis V., Prichard I. (2025). A Pilot Evaluation of the Acceptability and Feasibility of, and Preliminary Outcomes from, the Embrace Kids Classroom Program among Australian Pre-Adolescents. Body Image.

[62] Ciao A.C., Brown T.A., Levine M. (2024). Future Directions for Equity-Centered Body Image and Eating Disorders Prevention Work. Eat. Disord..

