# Supplementary material for: A Scoping Review of Eating Disorder Prevention and Body Image Programs Delivered in Australian Schools
Source: Nutrients. 2025 Jun 26;17(13):2118. doi: 10.3390/nu17132118 (PMC12251236; doi:10.3390/nu17132118)
Supplement: Supplementary file 1 [file nutrients-17-02118-s001.zip › nutrients-3710382-supplementary/Nutrients Supp files/Appendix A- Search Strategy.pdf]

Appendix A: Search Strategy

APA PsycINFO Search Strategy

1. eating disorder*.mp. or exp Eating Disorders/
2. binge eating.mp. or exp binge eating/
3. muscle dysmorphi*.mp. or exp muscle dysmorphia/
4. (other specified feeding and eating).mp.
5. emotional eating.mp. or exp emotional eating/
6. exercise dependence.mp. or exp exercise dependence/
7. body image.mp. or Body Image/
8. 1 or 2 or 3 or 4 or 5 or 6 or 7
9. early intervention.mp. or exp Early Intervention/
10. prevent*.mp. or exp Prevention/
11. universal prevention.mp.
12. selective prevention.mp.
13. 9 or 10 or 11 or 12
14. exp Schools/ or school*.mp.
15. 8 and 13 and 14
16. limit 15 to english language

Scopus
(TITLE-ABS-KEY ("eating disorder*" OR bulimi* OR anorexi* OR "body image" OR "binge* eating" OR pica OR "avoidant restrictive food intake" OR "body dissatisfaction" OR "muscle dysmorphi*" OR "emotional eating" OR "exercise dependence") AND TITLE-ABS-KEY ("early intervention" OR prevent* OR "universal prevention" OR "selective prevention") AND TITLE-ABS-KEY (school*)) AND (LIMIT-TO (LANGUAGE , "English"))

Embase Classic <1947 to 1973> Part 1 of 2
Embase <1974 to 2025 February 11>

1 eating disorders.mp. or exp eating disorder/
2 binge eating disorder/ or binge eating.mp.
3 body image.mp. or exp body image/
4 (other specified feeding and eating).mp.
5 early intervention.mp. or early intervention/
6 prevention/ or prevent*.mp.
7 universal prevention.mp.
8 selective prevention.mp.
9 schools.mp. or exp school/
10 exercise dependence.mp.
11 1 or 2 or 3 or 4 or 10
12 5 or 6 or 7 or 83665941
13 9 and 11 and 12 862
14 limit 13 to english language 799

MEDLINE

Ovid MEDLINE(R) ALL <1946 to February 11, 2025>

1	eating disorders.mp. or exp eating disorder/
2	binge eating disorder/ or binge eating.mp.
3	body image.mp. or exp body image/
4	(other specified feeding and eating).mp.
5	early intervention.mp. or early intervention/
6	prevention/ or prevent*.mp.
7	universal prevention.mp.
8	selective prevention.mp.
9	schools.mp. or exp school/
10	exercise dependence.mp.
11	1 or 2 or 3 or 4 or 10
12	5 or 6 or 7 or 8
13	9 and 11 and 12
14	limit 13 to english language
